# 获得性血友病A诊断与治疗中国指南（2021年版）

**DOI:** 10.3760/cma.j.issn.0253-2727.2021.10.001

**Published:** 2021-10

**Authors:** 中华医学会血液学分会血栓与止血学组 中国血友病协作组

一、概述

获得性血友病A（Acquired Hemophilia A, AHA）是一种由于循环血中出现抗凝血因子Ⅷ（FⅧ）自身抗体导致FⅧ活性（FⅧ∶C）降低的获得性出血性疾病。其特点为既往无出血史和无阳性家族史的患者出现自发性出血或者在手术、外伤或侵入性检查时发生异常出血。出凝血筛查以孤立性活化的部分凝血活酶时间（APTT）延长为特征。

AHA的年发病率约为1.5/100万，可发生于男女各年龄段，两个发病高峰分别为育龄女性的围产期及60岁以上人群，儿童罕见[1]–[3]。大约有50％的AHA患者可以发现病因或基础疾病，如自身免疫性疾病、恶性肿瘤、药物引起、感染等，1％～5％的患者发生于妊娠期或产后1年内[3]–[8]。本病的出血表型具有异质性，可有严重出血或者轻微出血甚至没有出血表现。患者就诊时的临床特征无法预测病程中是否会发生严重出血事件[2],[5],[9]。早期报道本病的死亡率可高达42％，近年来报道的死亡率不超过12％，中国获得性血友病登记（CARE）研究中死亡率为6.7％，主要死亡原因包括出血、基础疾病以及继发于免疫抑制治疗（IST）的严重感染等[10]–[11]。AHA治疗成功的关键在于及时诊断、及早给予恰当的治疗。

为了规范国内同行的诊疗行为，并为有关部门制定政策提供依据，我们先后制定了相关的中国专家共识/指南[12]–[13]。近年来，国内外有多项前瞻性登记研究结果发表，如CARE研究、欧洲GTH研究等，对于AHA的诊疗提供了新的依据[5],[14]–[15]。因此，有必要基于更高等级的最新证据对指南进行更新，为AHA诊治提供恰当的临床指导。

二、推荐等级

根据GRADE方法[16]，本指南推荐等级如下：

1级推荐：相当于“指南推荐”，代表该推荐对患者的安全性及获益明显高于风险和负担。1B级：该推荐至少有一项观察性或干预性研究的数据支持，且该推荐在大多数情况下适用于大多数患者；1C级：该推荐缺乏此类证据支持，但是仍然对患者的安全或获益很重要。

2级推荐：相当于“指南建议”，用于表示较弱的推荐，该建议可能会随着更新证据的出现发生改变。2B级：病例登记或研究数据支持该建议；2C级：无前述数据支持。

三、诊断

由于AHA具有罕见、突发及出血异质性大的特点，并且有时患者首诊并非在血液科而导致诊断延迟，因此国内外对于本病的认识均有待提高。CARE研究显示患者首次出血至确诊所需中位时间为30 d，就诊时严重出血的患者占60.9％[5]。因此，早期诊断有助于及时选择合适的止血方案、预防严重出血并及时清除抗体以恢复FⅧ∶C。

遇到以下情况时需要考虑本病的诊断：①既往无出血史的非血友病患者（尤其是老年人或者产后）出现自发性出血或外伤、有创操作后发生与预期不符的过度出血，合并不能解释的孤立性APTT延长（1B级）；②术前发现不能解释的孤立性APTT延长（1C级）[10]。

（一）临床表现

主要临床表现是近期急性出血，多为自发性，也可发生在手术/侵入性检查后，既往无出血史，无出血性疾病家族史。最常见的出血部位是皮下出血（约80％），肌肉出血次之（约40％），其他出血部位有泌尿生殖系、胃肠道、腹膜后和颅内出血等，关节出血少见[1]–[2],[5]。出血的危害取决于出血部位及出血量，颅内出血、咽喉部出血及胃肠道等部位出血可能危及生命，前臂或下肢出血导致骨筋膜室综合征、髂腰肌出血损伤股神经时具有很高的致残性，部分出血严重病例伴有贫血及血肿引致相关并发症，有时深部出血而皮肤表面并无瘀斑而难以及时判断为出血。少数患者没有出血表现，因其他原因检查凝血功能发现孤立性APTT延长而就诊[4]–[5]。

（二）实验室检查与诊断

疑似AHA的患者需要进行APTT混合血浆纠正试验进行抑制物筛查，推荐FⅧ∶C检测及抑制物定量来确诊（1B级）。如果同时伴有凝血酶原时间（PT）延长等，需要排除其他原因，如狼疮抗凝物（LA）、同时服用抗凝药物等。本指南中APTT延长的定义为超过本实验室的正常参考范围上限，或超过当天本实验室正常对照值10 s以上。通过建立简化的诊断路径（图1），让临床医师能够快速考虑并及早诊断本病，并选择恰当的治疗。

**图1:**
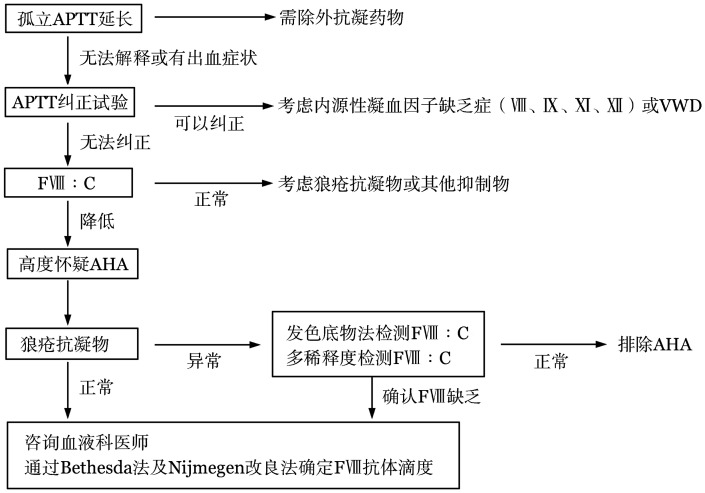
获得性血友病A（AHA）诊断路径 FⅧ：凝血因子Ⅷ；FⅧ∶C：凝血因子Ⅷ活性；VWD：血管性血友病

1. APTT混合血浆纠正试验[13],[17]：确定APTT延长后应进行正常血浆混合纠正试验，简称APTT纠正试验（2B级）。即正常血浆和患者血浆按1∶1混合后，于即刻和37 °C孵育2 h后再分别测定APTT，并与正常血浆和患者本身的APTT进行比较，若不能纠正应考虑可能存在抑制物[13]。本指南定义的APTT纠正是指超过正常混合血浆5 s以内（或延长<15％）或在实验室正常参考范围内；本指南定义的APTT不纠正是指超过正常混合血浆5 s以上（或延长>15％）或高于实验室正常参考范围。FⅧ抑制物特点是时间及温度依赖性，大多数抗体（尤其是较弱抗体）可以即刻完全或部分纠正，孵育2 h后不能纠正，高滴度抑制物可表现为即刻不能纠正。由于采用的试剂、仪器不同，每个实验室需根据自身的经验建立相应的规范和判读标准。纠正试验用于区分凝血因子缺乏或抑制物的存在，由于缺乏标准化，因此不能单独用来确定或者排除AHA的诊断，需要同时进行FⅧ∶C、FⅧ抑制物定量和LA检测，以尽快确诊[13],[17]。

2. 凝血因子活性检测：单一FⅧ∶C降低提示可能为AHA，需要除外遗传性血友病A、血管性血友病（VWD）和获得性VW综合征（AVWS）（1B级）。少数患者所有内源性凝血因子活性都降低，可能是由于FⅧ抑制物消耗底物血浆中FⅧ所致的假象。解决的方法是将患者血浆进行一系列稀释后再检测相应的凝血因子活性，其他凝血因子活性随着稀释比例增加而逐渐升高，但是FⅧ∶C变化不大。LA由于抑制依赖磷脂凝血过程而导致APTT延长，也可能会造成一期法检测内源性凝血因子活性降低的假象，但所有受影响的凝血因子活性随着稀释比例增加而逐渐升高。

3. 抑制物的定量[18]：确诊AHA必须测定抑制物滴度，常用的检测方法为Bethesda法及Nijmegen改良法。将不同稀释度的患者血浆与正常血浆等量混合，37 °C孵育2 h，测定残余FⅧ∶C。能使正常血浆FⅧ∶C减少50％时，则定义为FⅧ抑制物的含量为1个Bethesda单位（BU），此时患者血浆稀释度的倒数即为抑制物滴度，以BU/ml血浆表示。抑制物滴度≥0.6 BU/ml则为阳性。在Bethesda方法基础上改良的Nijmegen方法可以增加抑制物滴度较低时（<1 BU/ml）的特异性及敏感性[18]。

Bethesda法及Nijmegen改良法设计的初衷是为了检测遗传性血友病A患者中出现的针对FⅧ的同种抗体，这类抗体一般呈线性的1型动力学特征。AHA患者的FⅧ抗体则多表现为复杂的、非线性的2型动力学特征，即快速灭活期后平台期，有剩余FⅧ，滴度与稀释度不呈正比，因此以上方法可能并不能准确估计抑制物的真实效价，建议选用残余FⅧ∶C最接近50％的稀释度计算抑制物滴度（2B级）。为了避免AHA患者体内残余FⅧ对抑制物检测的干扰，尤其是FⅧ∶C>5％的患者，推荐采用热灭活方法（如56 °C 30 mins）灭活血浆中FⅧ，再进行检测，以增加抑制物检测的准确性及敏感性（1B级）。

（三）鉴别诊断

1. 血友病A伴抑制物：血友病A伴抑制物是血友病A患者接受FⅧ制剂治疗后产生的同种抗体，可完全灭活外源性FⅧ，多发生于重型患者，轻型和中型间患者较少。血友病A患者多有自幼反复、自发性出血史，以关节和肌肉出血、关节畸型为特点。约2/3有家族史，符合Ⅹ连锁隐性遗传规律。确诊血友病A患者在输注FⅧ制剂预防治疗时出血频率较前增加或按需治疗时止血效果不佳时，应怀疑抑制物的发生。初次拟诊的血友病A患者，尤其是轻型和中间型患者，在替代治疗前需检测FⅧ抑制物，以除外AHA。

2. 其他获得性凝血因子缺乏症：引起孤立性APTT延长者还见于其他内源途径的因子（FⅨ、FⅪ、FⅫ）及VWF缺乏，可通过相应的凝血因子及抑制物检测进行鉴别。需要指出的是，其他因子抑制物（如FⅤ）在高滴度时也会干扰其他因子一期法活性检测结果，需注意鉴别，方法可采用前述的稀释法。

3. LA：由于可以抑制磷脂功能，LA可表现为APTT延长（常见）和PT延长（少见）且不能被正常血浆纠正。LA引起的APTT延长一般为非时间依赖性，少数（10％～15％）可表现为时间依赖性。因此，时间依赖性的抑制物特性并不能完全区分FⅧ抑制物和LA。利用加入补充外源磷脂能够缩短或纠正APTT的特点，通过各种依赖磷脂的试验如稀释的蝰蛇毒试验（dRVVT）等证实LA的存在。

LA引起APTT延长的特点可导致体外试验中内源性凝血因子用一期法检测时活性“降低”的假象。发色底物法一般对LA不敏感；可用来鉴别[19]。例如一期法检测FⅧ∶C降低，而发色底物法检测FⅧ∶C正常时，可以排除AHA。由于国内发色底物法检测FⅧ∶C未常规应用，可以采用多个稀释度样本检测活性的方法鉴别这种假性减少。对于疑难复杂病例，ELISA检测FⅧ抗体可鉴别FⅧ抑制物和LA，这种检测较少开展。需要注意少数患者可同时出现抗FⅧ的自身抗体和LA。临床上，LA患者以血栓表现为主，很少发生出血（出血多见于血小板计数明显降低或伴有凝血因子缺乏时）。

四、治疗

AHA的治疗应在有经验的中心开展，或尽快转诊或寻求专家建议，以及时给予恰当的治疗。本病的治疗原则包括：去除诱因及治疗原发病、及时治疗及预防出血和尽早开始IST以清除FⅧ抑制物。

（一）去除诱因及治疗基础疾病

伴有肿瘤、皮肤病、感染等基础疾病患者应积极处理原发病，伴有自身免疫系统疾病（如结缔组织病、免疫性血小板减少症等）的患者，在选择IST方案前需要充分了解并考虑既往治疗史；药物相关AHA应脱离药物接触；部分患者无需特殊处理（如围产期女性）；对于起始没有发现基础疾病的患者，病程中需注意可能病因的显现，尤其一线及二线治疗均无效或反复复发时，需要继续查找潜在病因并处理。

（二）止血治疗

1. 止血治疗原则：确诊后应立即采取措施预防发生严重出血。应避免手术、有创操作等，如无法避免，应在有经验的中心或专家指导下，预防性应用旁路途径止血药物后完成（1B级）；发生肌肉血肿时尽量避免手术切开，以防止发生难以控制的出血，及时给予止血治疗，避免发生骨筋膜室综合征；由有经验的人员进行静脉穿刺并减少穿刺次数[10]。

止血药物治疗以控制患者急性出血为首要目标。但是需注意血栓形成的风险，尤其是老年患者或伴有血栓形成危险因素（有血栓发生史、持续制动、卧床等）。

由于抑制物滴度和FⅧ∶C与出血的严重程度相关性差[1],[4]，因此，止血治疗策略的制订应根据患者出血的严重程度，而不是抑制物滴度或残留FⅧ∶C（1B级）。

如果患者无明显出血或仅有局部皮肤瘀斑，只需密切观察并给予清除抑制物治疗，不需要特殊的止血治疗。对于腹膜后和咽后间隙出血、肌肉出血、颅内出血、消化道出血、泌尿道出血、肺出血和术后出血以及多部位出血等应予积极止血治疗。止血治疗选择见图2。

**图2:**
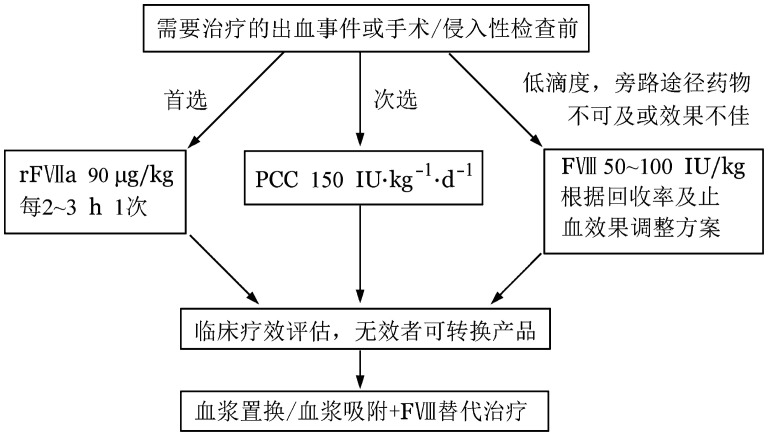
获得性血友病A止血治疗选择 rFⅦa：重组活化人凝血因子Ⅶ；PCC：凝血酶原复合物；FⅧ：凝血因子Ⅷ

2. 旁路途径药物止血治疗：旁路途径药物包括重组活化人凝血因子Ⅶ（rFⅦa）和活化凝血酶原复合物（aPCC）。aPCC未在国内上市，一般使用凝血酶原复合物（PCC），但是PCC的疗效仅基于回顾性研究及登记研究数据，缺乏对照研究结果支持[5]。

（1）rFⅦa：rFⅦa在多数研究中的止血有效率>90％，血栓栓塞事件发生率为0～2.9％[20]–[21]。CARE研究中，rFⅦa中位止血时间为5（2.5，6.75）h，显著优于PCC及FⅧ制剂[5]。rFⅦa单次剂量为90 µg/kg，也有研究者尝试减低剂量应用[5]。

推荐在临床判断出血事件需要止血治疗时，尽快给予rFⅦa 90 µg/kg每2～3 h 1次至出血控制（1B级）；如24 h后止血效果不佳，考虑转换其他止血药物（2C级）。

（2）PCC：CARE研究中，PCC单药止血有效率为84.6％，中位止血时间为72（42, 317）h，血栓事件发生率为8.8％[5]。在使用PCC过程中应注意监测血栓事件的发生。根据PCC产品中FⅦ含量的差异，PCC可分为三因子（FⅦ含量低）和四因子（FⅦ含量高）产品，目前尚无两种类型PCC疗效差异的研究数据。

当无法使用rFⅦa时，建议使用PCC止血，剂量一般不超过150 IU·kg^−1^·d^−1^，分次使用（2B级）。

（3）旁路途径药物联合应用：世界血友病联盟建议遗传性血友病A伴抑制物患者出血时，如应用单一旁路途径药物效果不佳，可以序贯使用rFⅦa及aPCC，如rFⅦa 90 µg/kg及aPCC 50 IU/kg间隔3 h 1次（rFⅦa及aPCC每日4次）交替[22]。该方案在AHA出血治疗中缺乏研究，且国内无aPCC产品供应。如患者出现难以控制的出血且rFⅦa或PCC效果均不佳时，综合权衡血栓事件并发症风险后，再谨慎选择序贯应用rFⅦa及PCC，使用过程中需严密监测血栓事件的发生。此外，如由于药物可及性或经济原因无法持续应用rFⅦa，可以考虑与PCC序贯使用。

3. FⅧ浓缩剂：即使在低滴度患者中，抑制物滴度也无法准确预测AHA患者输注FⅧ后的增量回收率，FⅧ∶C水平也不能预测止血疗效。EACH2及CARE研究中，FⅧ浓缩剂止血效果均低于旁路途径药物。CARE研究中，FⅧ制剂单药止血有效率为34.4％，中位止血时间为250（202, 306）h[5]。

建议仅在无法获得旁路途径药物或其疗效不佳且患者为低滴度抑制物时使用高剂量FⅧ止血。首次给予高剂量FⅧ（50～100 IU/kg），在输注后检测FⅧ增量回收率，并结合临床疗效调整用药剂量及间隔，以达到预期止血疗效。如疗效不佳，应及时更换其他止血药物（1B级）。

4. 1-去氨基-8-D-精氨酸加压素（DDAVP）：DDAVP在部分滴度<2 BU/ml且FⅧ∶C>5％的AHA患者中可能有一定疗效，一般剂量为每次0.3 µg/kg[9]。DDAVP有可能发生水肿、心衰、持续低钠血症和抽搐等不良反应。妊娠患者及2岁以下儿童禁用。老年患者多伴有心血管疾病，应用时须谨慎。对于AHA患者，仅在无其他选择且出血轻微时，经慎重评估后应用DDAVP止血。

5. 抗纤溶及其他药物：抗纤溶药物（氨甲环酸及氨基己酸等）可作为除泌尿系出血以外其他部位出血的辅助治疗。局部应用抗纤溶药物可作为黏膜等部位出血的替代方法。抗纤溶药物与旁路制剂的联用有增加血栓事件的风险，在使用中需要注意观察血栓事件的实验室检查及临床体征。应避免PCC与抗纤溶药物同时使用，如必须使用建议间隔6 h以上，以降低血栓事件风险。

双特异性抗体艾美赛珠单抗用于AHA的报道逐渐增多，由于血药浓度达峰需要数周，只能用于预防出血，不能用于治疗急性出血。在一项包含12例患者的研究中应用的方案为3 mg/kg皮下注射每周1次，共2～3次，随后1.5 mg/kg每3周1次，以预防出血[23]–[24]。

人工辅助凝血酶或者纤维胶可用于某些部位出血，如鼻出血、口腔溃疡、皮肤出血和外科手术部位等。

6. 止血疗效评估：止血疗效主要依靠临床综合评估，如患者的主诉、出血部位的症状及体征、血红蛋白水平、红细胞压积及影像学变化（1B级）。目前没有经过验证的实验室检查可以用来监测旁路途径药物止血效果。可常规监测FⅧ∶C，但与止血疗效并不一定一致。

（三）抑制物清除

1. 一般原则：AHA的IST方案缺少随机对照研究，但是近年来发表的登记注册研究结果为预后分层及个体化治疗提供了数据支持[25]。

所有患者在确诊后应立即采取IST以清除FⅧ抑制物，恢复FⅧ∶C（1B级）。在IST方案制定及实施过程中，应注意监测针对可能出现的并发症如骨髓抑制、糖皮质激素相关不良反应等，以避免继发感染等不良事件发生。尤其对于基础疾病较多的老年AHA患者，在积极治疗原发病的基础上，应权衡快速清除抑制物以降低出血风险和IST不良反应之间的利弊后制定IST方案（1B级）。

2. 一线治疗：表1中列举了AHA患者IST一线方案。CARE研究中糖皮质激素单药、糖皮质激素联合环磷酰胺及给予利妥昔单抗方案的中位部分缓解时间分别为56、42及42 d。

糖皮质激素在患者获得缓解或用至6周后逐渐减停。有大剂量地塞米松用于AHA一线治疗的报道，但并不能改善感染率及死亡率[27]。环磷酰胺除上述方案外，亦可200 mg隔日1次静脉给药。

3. 二线治疗及其他治疗：AHA患者经过一线治疗3～5周后抑制物滴度无明显下降或FⅧ∶C较基线值无明显上升时考虑给予二线治疗。

对于糖皮质激素单药患者，二线治疗可以加用环磷酰胺或者利妥昔单抗，剂量见表1。对于糖皮质激素联合环磷酰胺或者利妥昔单抗患者，二线治疗可换用没有使用过的药物（利妥昔单抗或环磷酰胺）（2B级）。

**表1:** 获得性血友病A免疫抑制治疗一线方案

推荐一线方案	推荐剂量	说明	注意事项
糖皮质激素单药	泼尼松1mg·kg^−1^·d^−1^口服或等效剂量其他类型糖皮质激素口服或静脉给药，疗程一般不超过6周，逐渐减量至停用	预后不良组患者在3周内有效的可能性较小	监测潜在风险（高血糖、感染、骨质疏松、股骨头坏死及精神疾病等）
糖皮质激素联合环磷酰胺	糖皮质激素同上；环磷酰胺1.5～2mg·kg^−1^·d^−1^，静脉或口服给药，疗程一般不超过6周	较糖皮质激素单用起效快、缓解率高	环磷酰胺潜在风险（骨髓抑制、继发感染等）
糖皮质激素联合利妥昔单抗[26]	糖皮质激素同上；利妥昔单抗375mg/m^2^每周1次，静脉给药，最多4次或100mg每周1次×4次	不推荐单药，除非患者有其他免疫抑制药物禁忌症	原则上禁用于活动性乙型肝炎患者，注意感染监测及预防

一线及二线治疗均无效时，可尝试其他免疫抑制剂，如霉酚酸酯、硫唑嘌呤、长春新碱、环孢素A和他克莫司等[25]。蛋白酶体抑制剂也有成功用于AHA的报道[28]。对于一些难治复发的患者，可以重复之前有效的药物，或通过临床试验探索更多药物用于AHA的有效性及安全性评价。

对于长期治疗无效或复发的患者，需再次寻找肿瘤、自身免疫疾病等病因证据（2C级）。

多项研究显示，大剂量静脉注射免疫球蛋白（IVIG）在AHA中疗效不佳，因此不推荐以清除抗体为目的使用IVIG（1B级）[1],[10],[29]。

研究显示血浆置换或者免疫吸附法在难治性出血事件或需要外科干预等特殊情况下应用可快速去除血浆中的抑制物并补充FⅧ，以达到有效止血，但是其无法持续清除抑制物[26]。

4. 个体化治疗：基于GTH研究，2020年的AHA诊疗国际推荐将AHA患者IST疗效分为良好组及不良组，良好组为FⅧ∶C≥1％且抑制物滴度≤20 BU/ml；不良组为FⅧ∶C<1％或抑制物滴度>20 BU/ml。良好组一线方案推荐给予糖皮质激素单药，不良组一线方案推荐糖皮质激素联合环磷酰胺或利妥昔单抗。如3～4周无反应，则给予二线方案[10],[25]。

推荐条件允许时根据预后分层及患者体能状况制定IST方案（2B级）。

两组患者对一线方案未能达到部分缓解标准，但FⅧ∶C和抑制物滴度持续改善的患者，建议适当延长当前治疗方案至4～6周（2B级）。

5. 妊娠相关AHA的IST治疗：环磷酰胺对于妊娠期及哺乳期AHA患者并不安全，IST方案首选糖皮质激素单药治疗，也有利妥昔单抗用于该类患者治疗的报道。这部分患者有一定的自发缓解率，但是IST治疗达完全缓解的时间比其他类型AHA长[6],[10]。

6. 抑制物清除疗效判断：本指南推荐标准如下[5],[25]：完全缓解：抑制物滴度<0.6 BU/ml、FⅧ∶C≥50％，免疫抑制剂停用或恢复至发病前剂量。部分缓解：FⅧ∶C≥50％、抑制物滴度≥0.6 BU/ml，止血治疗结束后24 h无新发出血。无效：FⅧ∶C<50％，抑制物滴度≥0.6 BU/ml，伴或不伴活动性出血。复发：完全缓解或部分缓解患者随访中发生FⅧ∶C<50％且抑制物滴度≥0.6 BU/ml。

7. 治疗过程监测：建议在IST期间，每周检测1次FⅧ∶C、抑制物滴度、血常规等，以评估疗效及评估IST可能的并发症。其他伴随疾病的监测，取决于临床评估需要，尽量减少可能引起出血的检查。

五、随访

AHA患者在完全缓解后应继续随访监测FⅧ∶C，最初6个月内每月检测1次；6～12个月时每2～3个月检测1次；第2年每半年检测1次；此后可酌情延长检测间隔。可根据临床需求复查FⅧ抑制物定量。
